# Identification of Yeast Mutants Exhibiting Altered Sensitivity to Valinomycin and Nigericin Demonstrate Pleiotropic Effects of Ionophores on Cellular Processes

**DOI:** 10.1371/journal.pone.0164175

**Published:** 2016-10-06

**Authors:** Michaela Jakubkova, Vladimira Dzugasova, Dominika Truban, Lenka Abelovska, Ingrid Bhatia-Kissova, Martin Valachovic, Vlasta Klobucnikova, Lucia Zeiselova, Peter Griac, Jozef Nosek, Lubomir Tomaska

**Affiliations:** 1 Department of Biochemistry, Comenius University in Bratislava, Faculty of Natural Sciences, Mlynska dolina, Ilkovicova 6, 842 15 Bratislava, Slovakia; 2 Department of Genetics, Comenius University in Bratislava, Faculty of Natural Sciences, Mlynska dolina, Ilkovicova 6, 842 15 Bratislava, Slovakia; 3 Institute of Animal Biochemistry and Genetics, Slovak Academy of Sciences, Department of Biochemistry of Biomembranes, Moyzesova 61, 900 28 Ivanka pri Dunaji, Slovakia; University of Leicester, UNITED KINGDOM

## Abstract

Ionophores such as valinomycin and nigericin are potent tools for studying the impact of ion perturbance on cellular functions. To obtain a broader picture about molecular components involved in mediating the effects of these drugs on yeast cells under respiratory growth conditions, we performed a screening of the haploid deletion mutant library covering the *Saccharomyces cerevisiae* nonessential genes. We identified nearly 130 genes whose absence leads either to resistance or to hypersensitivity to valinomycin and/or nigericin. The processes affected by their protein products range from mitochondrial functions through ribosome biogenesis and telomere maintenance to vacuolar biogenesis and stress response. Comparison of the results with independent screenings performed by our and other laboratories demonstrates that although mitochondria might represent the main target for both ionophores, cellular response to the drugs is very complex and involves an intricate network of proteins connecting mitochondria, vacuoles, and other membrane compartments.

## Introduction

Membrane compartments of eukaryotic cells are highly dynamic structures undergoing dramatic morphological and physiological changes in response to variations in extracellular and intracellular environment. Defects in organellar dynamics often have fatal consequences, as documented by several examples of human diseases caused by deficiencies in cellular components involved in organelle biogenesis and morphogenesis [1–4]. For example, the dynamic nature of mitochondria is characterized by very rapid cycles of fusion, fission, and tubulation, governed by a wide range of proteins [5–7]. These processes are essential for maintenance of their heterogeneity, thus contributing to robustness on the level of cellular physiology [8]. Studies from the last two decades, combining microscopic techniques with biochemical and genetic approaches, have revealed the molecular nature of many players in mitochondrial dynamics. It is characterized by complex interconnections between mitochondrial shape, inheritance, biogenesis, as well as other processes, including interorganellar interactions or lipid metabolism (for review, see [5]).

Mitochondrial morphology and dynamics can be substantially compromised also by disturbed ion homeostasis. Despite a relatively low permeability of the inner mitochondrial membrane (IMM) for inorganic ions, an electrophoretic influx of cations (mainly K^+^ and Mg^2+^), accompanied by osmotic water leakage, is driven by the membrane potential, ΔΨ [9–11]. Several ion transporters operate in the IMM to prevent matrix swelling and thus maintain structural and functional integrity of the organelle. In yeast, there are five known mitochondrial cation transporters (for review, see [12]): the Mg^2+^ channels Mrs2p [13, 14] and Lpe10p [15], the Fe^2+^ transporters Mrs3p and Mrs4p [16], and the presumed K^+^/H^+^ antiporter component Mdm38p/Mkh1p, a homologue of mammalian LETM1, which is mutated in Wolf-Hirschhorn syndrome [17, 18]. Mitochondria of the Δ*mdm38* mutant display several impairments, including K^+^ accumulation, swelling, and formation of rings or lariat-like structures [17, 19], which illustrate close connection between mitochondrial shape and ion homeostasis.

Cations, in this case hydronium, are also key in the interorganellar interplay with important consequences for cell physiology and aging. During early mother-cell divisions, vacuolar acidity progressively declines, limiting lifespan of the mother cell by affecting mitochondrial functions [20]. According to the current hypothesis, the excess of cytoplasmic neutral amino acids (or other metabolites as well), which could not be transported to vacuoles due to the increased pH of their lumen, is catabolised in mitochondria. As a consequence, overloaded proton-dependent carrier proteins reduce ΔΨ at the IMM.

Our approach to study molecular mechanisms involved in the relationship between mitochondrial ion homeostasis and mitochondrial (or, more generally, organellar) morphology and dynamics was based on experimental results describing a selective effect of potassium ionophores nigericin and valinomycin on *S*. *cerevisiae* inner mitochondrial membrane [21–23]. Nigericin is a chemical K^+^/H^+^ antiporter which dissipates ΔpH but not ΔΨ on the IMM [24]. Valinomycin mediates K^+^ uniport across the IMM resulting in dissipation of the K^+^ gradient and subsequently in decrease of ΔΨ [25]. Kovac and colleagues have shown that both ionophores inhibit growth of *S*. *cerevisiae* on a non-fermentable carbon source [21–23]. When yeasts grow on glucose both ionophores induce formation of respiratory-deficient mutants. Further results of these authors supported the hypothesis that in yeasts both nigericin and valinomycin act preferentially on the IMM. The molecular basis of this preference remained, however, unclear. Previously, we observed that *S*. *cerevisiae* clones resistant to the presence of a single ionophore, either valinomycin or nigericin, arose spontaneously at a relatively high frequency (5x10^-5^) [26]. This observation indicates that a simple mutation within a probably broad gene repertoire may lead to changes allowing the cell to respire in the presence of the ionophore. Analysis of such mutants could provide new insights into mitochondrial ion homeostasis and other related processes.

Indeed, in our first genomic screen, based on transposon mutagenesis (“Tn-screen”), we found several ionophore-resistant mutants with changes on the level of mitochondria [27]. For example, the nigericin-resistant (Nig^R^) *mdm31* and *mdm32* clones (together with other mitochondrial distribution and morphogenesis mutants originally described and named by [28, 29]) contain giant mitochondria and frequently lose their mitochondrial DNA. Importantly, we also observed a decreased swelling ability of their mitochondria in isotonic solutions of potassium acetate (in spite of the presence of an exogenous K^+^/H^+^ antiporter) and suppression of their mitochondrial morphology defects by the addition of nigericin.

In a complementary genetic screen, we identified ionophore-resistant mutants among *S*. *cerevisiae* cells subjected to UV mutagenesis [26]. We isolated several valinomycin-resistant (Val^R^) and/or Nig^R^ mutants exhibiting peculiar phenotypic characteristics. For example, the mutant *valR1* (selectively resistant to valinomycin) is unable to grow on complex media, whereas its growth on synthetic media is indistinguishable from that of the parental strain. In contrast, the mutant *valR2* lost the ability to grow on complex media containing 0.2 M calcium cations, while Ca^2+^ at the same concentration did not inhibit growth of this strain on synthetic media [26]. Complex media-related growth defects had been observed in case of several mutants defective in ergosterol metabolism [30]. It is known that lipid composition plays an important role in mitochondrial morphogenesis and inheritance [31]. Interestingly, some of the ionophore-resistant mutants identified in our screens have indeed abnormal total cellular lipid profiles [26].

Our results also illustrate the important role of vacuoles in cellular response to the ionophores [26, 32]. The possibility of their involvement was first suggested by the fact that one of the transposon mutants resistant to nigericin had a defect in the vacuolar amino acid permease Vba1p [33]. In addition, one of the Val^R^ mutants obtained by UV-mutagenesis (*valR1*) had impaired vacuolar fusion [26]. We have found that nigericin induces rapid changes in vacuolar morphology and pH. Immediately after addition of nigericin vacuoles fused into one central organelle and, as indicated by neutral red staining, their pH increased along with cytoplasm acidification. In case of Nig^R^ mutants, these changes reversed after prolonged incubation ([32]; Truban, D., Bhatia-Kissova, I., Nosek, J., unpublished results). The effect of nigericin on vacuoles has been also demonstrated by several other studies. For example, it was shown that nigericin (and monensin) extended chronological lifespan in *Schizosaccharomyces pombe* by affecting vacuolar acidification. This effect was dependent on the presence of the vacuolar ATPase (V-ATPase) subunits Vma1 and Vma3 [34].

These observations, together with the variety of several ionophore-resistant mutants identified in previous screens from our [26, 27] and other [35] laboratories, indicated that although nigericin and valinomycin primarily affect ion distribution in mitochondria, their impact on the cell is more complex. By screening the haploid deletion mutant library covering almost all nonessential genes of *S*. *cerevisiae* we obtained a set of mutants resistant or hypersensitive to valinomycin and/or nigericin. These mutants were deficient in a wide repertoire of proteins involved in processes ranging from mitochondrial and vacuolar functions, through translation to stress responses indicating novel targets of these ionophores.

## Materials and Methods

### Cultivation media

Complex yeast extract—peptone media (YPD–with 2% glucose, YPG–with 3% glycerol) and synthetic media with 2% (w/v) glucose (SD), containing 0.67% (w/v) yeast nitrogen base supplemented with appropriate amino acids (30 mg/l) and nitrogen bases (40 mg/l), were prepared as described by [36]. The semi-synthetic glycerol (sSG) medium contained 0.67% (w/v) yeast nitrogen base without amino acids, 0.1% (w/v) yeast extract, 3% (v/v) glycerol supplemented with appropriate amino acids (30 mg/l) and nitrogen bases (40 mg/l). Stock solutions of inhibitors are listed in S1 Table.

### Genomic screen for mutants with altered sensitivity to ionophores

Deletion mutants in the BY4741 background (EUROSCARF, Germany, yeastdeletion.stanford.edu) and the control strains (wild-type BY4741, W303-1B Δ*mdm31* (resistant to nigericin, Nig^R^; [27]) and W303-1B Δ*rtc1* (resistant to valinomycin, Val^R^; [27])) were pre-grown on YPD + geneticin sulphate (G418) plates, then suspended in 200 μl YPD in 96-well microtiter plates, cultivated overnight (28°C, with constant shaking), and diluted 20-times in water (dilution optimized for distinguishing growth differences between the wild type, hypersensitive and resistant mutants). The suspensions were spotted in quadruplicates, with a pinning tool, onto five different sSG media, one supplemented only with the required amino acids and nitrogen bases, the others containing also either valinomycin (Applichem) or nigericin (Applichem), each at two concentrations; 0.5 mg/l or 15 mg/l (the effective concentrations of nigericin varied between the lots, so they were titrated for each new batch of the ionophore). The plates were photographed after 7-day cultivation at 28°C. A strain that exhibited no growth or markedly poorer growth on 0.5 mg/l ionophore compared to the control plate (sSG) in at least three spots of the quadruplicate was designated as hypersensitive. A strain growing on 15 mg/l ionophore in at least three spots of the quadruplicate was considered as resistant. The growth was evaluated visually and a strain was considered “growing” at a spot only when growing uniformly, thus numerous cases displaying only several independent colonies within a cell suspension drop were excluded. The candidate strains from this first round of selection were re-assayed at least twice by manual drop tests, where three decimal dilutions of each culture were pipetted on the same types of media, and only those retaining ionophore-related phenotype in more than a half of the tests were kept on the final list.

### Drug susceptibility test

The susceptibilities of the mutants to different compounds (oligomycin, antimycin A, ethidium bromide, cycloheximide, and fluconazole) were tested as described in [26]. Concentrations of drugs used for the assays are indicated in S1 Table.

### Gene ontology enrichment analysis

Sets of genes whose disruption led to altered ionophore sensitivity were verified for significantly enriched Gene ontology (GO) categories using the Functional Specification resource, *FunSpec*, with the *P* value cutoff of 0.001, with as well as without Bonferroni-correction for multiple testing [37]. These statistical analyses were performed taking into account all the features in *Saccharomyces* Genome Database (SGD) that have GO annotations. The same analysis restricted to the subset of nonessential genes deleted in the mutant library was not possible with this software, thus we took advantage of the *GO Term Finder* enabling to specify the background set of genes. We used the set obtained from the list of mutants from EUROSCARF (as the collection includes several mutants twice, the duplicated genes were omitted from the list). Otherwise, default settings were applied (including the *P* value cutoff of 0.01).

### Construction of Vba1-yEGFP3 expressing plasmid

The open reading frame (ORF) for vacuolar permease for basic amino acids (Vba1/YMR088c) was amplified from the genomic DNA of *S*. *cerevisiae* (strain T2-3D) by polymerase chain reaction (PCR) using the primers 5’-CCATCGATATGCAAACACTAGACGAGAC-3’ and 5’-CCATCGATAGAACTTGAACTACGTTTGT-3’. The PCR product was then digested with *Cla*I endonuclease and the 1.69 kbp fragment was inserted into the *Cla*I site of the plasmid vector pUG35 (provided by J.H. Hegemann, Heinrich Heine Universität, Düsseldorf, Germany) in frame with the coding sequence for yeast enhanced version of green fluorescent protein (yEGFP3). The resulting plasmid construct (named pDF01) expresses Vba1 tagged at its C terminus (i.e. Vba1-yEGFP3).

### Ergosterol synthesis mutants

Deletion of *ERG* genes (see Results) in the SCY325 strain was described by Valachovic et al. [38]. Primers used to construct and confirm *erg* deletions in BY4743 strain are listed in the S2 Table. For each *erg* deletion, sets of forward and reversed primer (frwERG#kanMX and revERG#kanMX, where # represents the number of the corresponding *ERG* gene) were used to amplify the *kanMX4* disruption cassette, which was then introduced into the wild type diploid strain BY4743. Each primer consists of 80 bp of a genomic sequence that flanks either the 5' or the 3' end of the ORF, and a sequence homologous to the *kanMX4* cassette. Deletions were verified from both sides of the deleted *ERG* gene. For the verification at the 5' end, a primer in the promoter region of the deleted gene (frwERG#chck) and a kanB primer that recognizes a sequence in the *kanMX4* cassette, were used in PCR reactions. In addition, the complete deletion of the *ERG* gene was confirmed by PCR amplification using a primer in the terminator region of the deleted gene (revERG#chck) and a kanC primer that recognizes a sequence in the *kanMX4* cassette. *erg* deletion mutants (Δ*erg2*, Δ*erg3*, Δ*erg4*, Δ*erg6* and Δ*erg28*) were isolated as G418 resistant spores after sporulation and tetrad dissection of BY4743 diploids. Specifically, tetrads were dissected onto YPD plates using a Singer micromanipulator (Singer MSM System 300, Singer Instruments) and then grown for 3 days at 30°C. Individual viable colonies were tested for G418 resistance on YPD containing 200 μg/ml G418 (Sigma-Aldrich) and for segregation of auxotrophic markers (*met15* and *lys2*) on SD medium without methionine or lysine, respectively.

### Isolation and analysis of sterols

Sterols were isolated as non-saponifiable lipids after hydrolysis in methanolic potassium hydroxid. Shortly, cells were broken by homogenization with glass beads and incubated in 3 ml of 60% KOH (w/v) in 50% methanol (v/v) for 2 hours at 70°C. Sterols were extracted twice with 3 ml of *n*-hexane and the combined extracts were dried under nitrogen stream. Lipid residues were dissolved in *n*-hexane and analyzed by reversed phase HPLC on Agilent 1100 instrument equipped with Eclipse XDB-C8 column (Agilent Technologies, USA), diode array detector (Agilent Technologies, USA), and Corona Charged Aerosol Detector (ESA Inc., USA). The sterols were eluted at 30°C with 95% methanol at a flow rate 1 ml.min^-1^. The identity of ergosterol was determined from the retention time of ergosterol standard and verified based n its characteristic UV-vis spectrum.

## Fluorescence Microscopy

The wild-type strain BY4741 and the transformants expressing fluorescent proteins Vba1-yEGFP3 [32] or mt-pHluorin [39] were grown at 28°C in sSG medium supplemented with appropriate amino acids and nitrogen bases. The overnight cultures (about 1x10^7^ cells/ml) were centrifuged (5 min, 1,800 *g*, room temperature) and the cells were resuspended in the same volume of fresh sSG medium. Organelles (vacuoles and mitochondria) in non-transformed cells were stained with 10 μM FM4-64 (Invitrogen) for 45 min followed by 60 min incubation in the fresh cultivation medium, 50 μg/ml neutral red (Merck) for 5 min, or 2.5 μg/ml DiOC_6_ (Sigma) for 45 min. Nigericin (15 mg/l) or valinomycin (15 mg/l) has been added to the samples (200 μl) of cell cultures and the cells were immediately observed using Olympus BX50 fluorescence microscope equipped with the appropriate filter set and digital camera DP70.

## Results

### Genome-wide screen identifies deletion mutants with altered sensitivity to valinomycin and/or nigericin

The yeasts *S*. *cerevisiae* are sensitive to valinomycin and nigericin when cultivated on the semi-synthetic glycerol containing (sSG) medium. To identify mutations modulating sensitivity to these ionophores we screened a collection of 4720 *S*. *cerevisiae* haploid deletion mutants on sSG medium containing either 15 mg/l (for identification of resistant (R) mutants) or 0.5 mg/l (for identification of hypersensitive (HS) mutants) of either ionophore. Although the threshold concentrations varied slightly from experiment to experiment, we chose concentrations of ionophores at which the wild type strain BY4741 was always inviable (15 mg/l), or always viable (0.5 mg/l).

**Fig 1:**
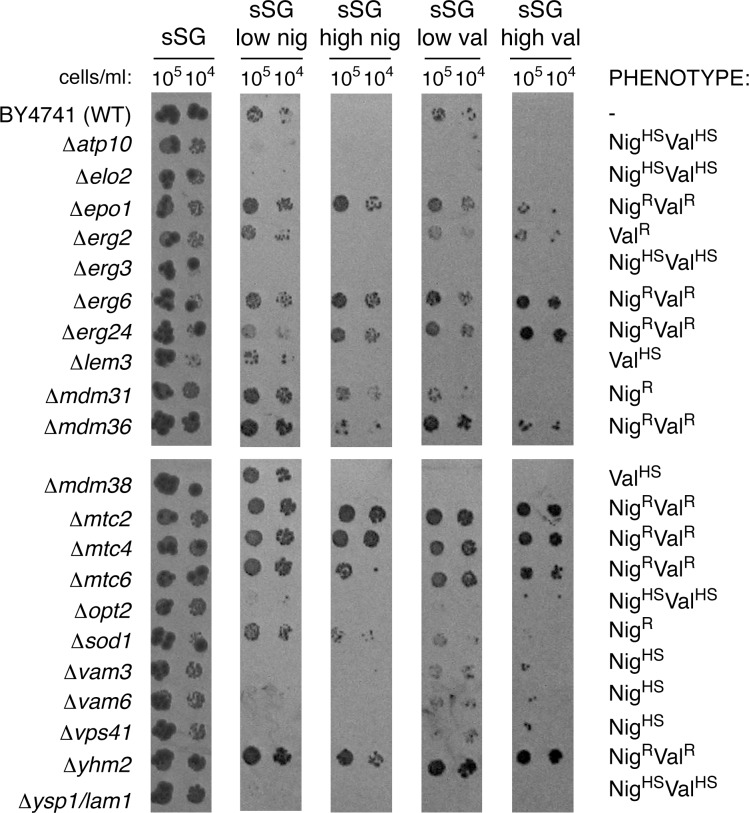
Examples of strains exhibiting different sensitivity to ionophores compared with the wild-type. Three microliters of the suspensions with the indicated concentrations of cells were spotted on the corresponding sSG media containing ionophores.

In the first round of selection, we identified 274 mutants, including 166 Val^R^, 28 Nig^R^, 2 Val^R^Nig^R^, 49 Val^HS^, 16 Nig^HS^, and 13 Val^HS^Nig^HS^. 127 of them remained on the list after two additional drop tests on the same media (Table 1 and S3 Table). The examples of strains exhibiting different sensitivity to the ionophores are presented in Fig 1. Notably, the ionophore sensitivity of about half of the 127 mutants varied from experiment to experiment. In case of sensitive strains, we often observed formation of microcolonies. We also frequently observed faster growing microcolonies in macrocolonies of the resistant strains after a prolonged cultivation in the presence of ionophores (data not shown). Most of the 127 mutants were tested for pleiotropic drug resistance (PDR) and none exhibited PDR phenotype (data not shown).

**Table 1:** Summary of the number of strains in each ionophore-resistance-related category identified in this study.

Category	# mutants
Nig^R^	6
Nig^HS^	14
Val^R^	64
Val^HS^	20
Val^R^ Nig^R^	8
Val^HS^ Nig^HS^	15
Σ	**127**

### Gene Ontology analysis of the genes involved in altered ionophore sensitivity

To analyse possible relationships between the genes whose deletions result in changes of sensitivity to valinomycin and/or nigericin, we performed a Gene Ontology analysis. As the number of strains in all individual categories, except for Val^R^, was too low for significant (*P* value < 0.001) statistical analyses, we employed the *FunSpec* software [37] (http://funspec.med.utoronto.ca/) for the analysis of all 127 identified genes. The over-represented categories included vacuolar functions (including microautophagy), lipid and sterol biosynthesis, and protein synthesis (represented mostly by protein components of the cytoplasmic ribosomal small subunit) (Table 2). In addition, according to Yeast Fitness Data database, a relatively large proportion of the mutants (41/127, >30%) are characterized as "slow growers“. 11 genes are directly annotated to root term ‘molecular function unknown’ and 15 are “dubious ORFs“.

**Table 2:** Functional groups enriched in the screen for mutants with altered sensitivity to ionophores.

Category and function (*P* value*)	Genes	k	f
GO biological process:			
vacuole fusion, non-autophagic (1.5E-05)	*PEP3****(V***^***HS***^***)***, *VAM6****(N***^***HS***^***)***, *VPS41****(N***^***HS***^***)***, *VAM7****(N***^***HS***^***)***, *VAM3****(N***^***HS***^***)***, *OPT2****(V+N***^***HS***^***)***	6	29
lipid biosynthesis (5.4E-05)	*ERG6****(V+N***^***R***^***)***, *ERG2****(V***^***R***^***)***, *ERG24****(V+N***^***R***^***)*,** *FEN1****(V***^***HS***^***)***, *SUR4****(V***^***HS***^***)***, *CRD1****(V+N***^***HS***^***)***, *ERG3****(V+N***^***HS***^***)***	7	52
regulation of SNARE complex assembly (1.3E-04)	*PEP3****(V***^***HS***^***)*,** *VAM6****(N***^***HS***^***)***, *VPS41****(N***^***HS***^***)***	3	6
sterol biosynthesis (2.0E-04)	*ECM22****(V***^***R***^***)***, *ERG6****(V+N***^***R***^***)***, *ERG2****(V***^***R***^***)***, *ERG24****(V+N***^***R***^***)*,** *ERG3****(V+N***^***HS***^***)***	2	29
response to reactive oxygen species (3.7E-04)	*SOD1****(N***^***R***^***)***	1	2
piecemeal microautophagy of nucleus ((3.7E-04)	*ATG15****(V***^***R***^***)***, *VAM6****(N***^***HS***^***)***, *VPS41****(N***^***HS***^***)***, *VAM7****(N***^***HS***^***)***, *VAM3****(N***^***HS***^***)***	5	23
GO Cellular Component:			
HOPS complex (1.3E-04)	*PEP3****(V***^***HS***^***)*,** *VAM6****(N***^***HS***^***)***, *VPS41****(N***^***HS***^	3	6
cytosolic small ribosomal subunit (1.7E-04)	*RPS16B****(V***^***R***^***)***, *RPS11A****(V***^***R***^***)***, *RPS27B****(V***^***R***^***)***, *RPS4A****(V***^***R***^***)***, *RPS16A****(V***^***R***^***)***, *RPS10B****(V***^***R***^***)***, *RPS19B****(V***^***R***^***)***	7	62
MIPS Functional Classification:			
superoxide metabolism	*SOD1****(N***^***R***^***)***	1	2
tetracyclic and pentacyclic triterpenes metabolism	*ERG6****(V+N***^***R***^***)***, *ERG2****(V***^***R***^***)***, *ERG24****(V+N***^***R***^***)*,** *ERG3****(V+N***^***HS***^***)***, *OSH3****(V+N***^***HS***^***)***	5	36
MIPS Protein Complexes:			
cytoplasmic ribosomal small subunit (9.8E-05)	*RPS16B****(V***^***R***^***)***, *RPS11A****(V***^***R***^***)***, *RPS27B****(V***^***R***^***)***, *RPS4A****(V***^***R***^***)***, *RPS16A****(V***^***R***^***)***, *RPS10B****(V***^***R***^***)***, *RPS19B****(V***^***R***^***)***	7	57
Vacuolar assembly complex (3.7E-04)	*VAM6****(N***^***HS***^***)***, *VPS41****(N***^***HS***^***)***	2	2
Vam3/Vam7 vacuolar t-SNARE complex (3.7E-04)	*VAM7****(N***^***HS***^***)***, *VAM3****(N***^***HS***^***)***	2	2
Yeast Fitness Data:			
Slow growers (2.7E-13)	*HCM1****(V***^***R***^***)***, *SRB8****(V***^***R***^***)***, *RPS11A****(V***^***R***^***)***, *REG1****(V***^***R***^***)***, *HPR1****(V***^***R***^***)***, *BUD26****(V***^***R***^***)***, *GET2****(V***^***R***^***)***, *SRB5****(V***^***R***^***)***, *RPS27B****(V***^***R***^***)***, *PIH1****(V***^***R***^***)***, *CTF8****(V***^***R***^***)***, *BUD19****(V***^***R***^***)***, *RPL39****(V***^***R***^***)***, *RPS4A****(V***^***R***^***)***, *MRT4****(V***^***R***^***)***, *DBP7****(V***^***R***^***)***, *RSA1****(V***^***R***^***)***, *YAR1****(V***^***R***^***)****ERG6****(V+N***^***R***^***)***, *RPS16A****(V***^***R***^***)***, *ERG2****(V***^***R***^***)***, *TMA23****(V***^***R***^***)***, *ERG24****(V+N***^***R***^***)***, *RPS19B****(V***^***R***^***)***, *FPK1****(V***^***R***^***)***, *RRP6****(V***^***R***^***)***, *SOD1****(N***^***R***^***)***, *MDM31****(N***^***R***^***)***,***)***, *PEP3****(V***^***HS***^***)***, *VRP1****(V***^***HS***^***)***, *OPI9****(V***^***HS***^***)***, *SUR4****(V***^***HS***^***)***, *SPT21****(V***^***HS***^***)***, *AKR1****(N***^***HS***^***)***, *YJL175W****(V+N***^***HS***^***)***, *ILM1****(V+N***^***HS***^***)***, *ERG3****(V+N***^***HS***^*)*, *CTK3****(V+N***^***HS***^***)***, *AEP1****(V+N***^***HS***^***)***, *TAF14****(V+N***^***HS***^***)***	40	616

* The *P* value reflects the probability that the observed annotation of the particular GO term to a group of genes occurs by chance [37] (http://funspec.med.utoronto.ca/cgi-bin/funspec); k, number of genes from the input cluster (127) in given category; f, number of genes total in given category; V^R^, valinomycin resistant; N^R^, nigericin resistant; V^HS^, valinomycin hypersensitive; N^HS^, nigericin hypersensitive; V+N^HS^, valinomycin and nigericin hypersensitive.

Next, we performed a manual examination of the GO categories summarized in SGD. Although in many cases the statistical significance was relatively low, this examination potentially revealed interesting co-occurring phenotypes (S3 Table). The most frequently associated phenotypes were decreased respiratory growth rate, decreased resistance to ethanol, and decreased resistance to altered pH. Interestingly, annotations of about 20% of Val^R^ and 12% of Nig^R^ mutants include decreased toxin resistance. The ionophore resistance is therefore not only generally unrelated to improved tolerance to toxic compounds, but can also be accompanied by increased vulnerability to other toxins.

Defects in mitochondrial organization and replicative cell aging appear as over-represented disturbed processes especially among Nig^R^ mutants. This high occurrence of mitochondrial organization defects is in line with abnormal mitochondrial morphology in almost 40% of Nig^R^ strains (e.g. Δ*mdm31*, Δ*mdm32*). On the other hand, about 40% of Nig^HS^ mutants have impaired vacuolar morphology and/or impaired autophagy, and sensitivity to CaCl_2_ (often associated with vacuolar defects).

Another category of mutants enriched in our screen were strains defective in sterol biosynthesis (Table 2). Interestingly, the Δ*erg24* mutant exhibiting Val^R^Nig^R^ phenotype was shown to be nonviable in other genetic backgrounds (such as W303). Such ambiguities may be caused either by a secondary suppressor mutation, a compensatory gain of a whole chromosome (such as the chromosome VII in case of Δ*erg4* in the original *S*. *cerevisiae* deletion library [40]), and/or other genetic changes in the EUROSCARF BY4741 strains.

**Fig 2:**
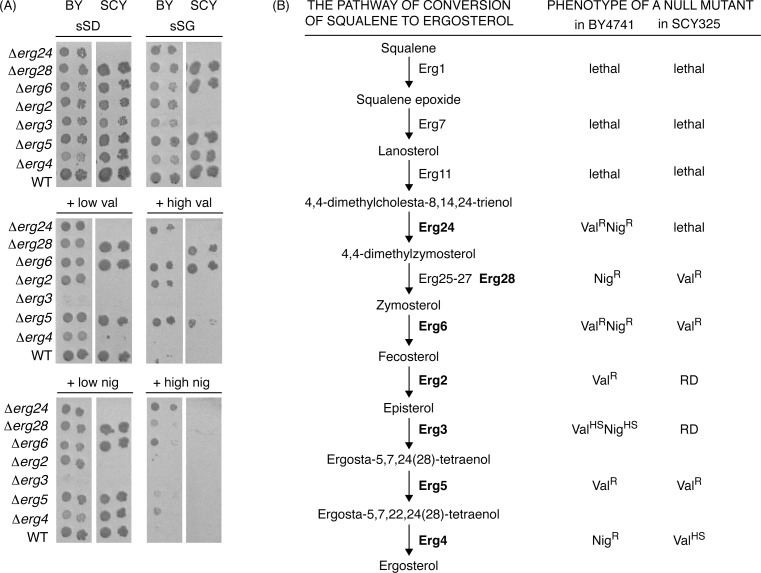
All viable *erg* mutants exhibit changes in sensitivity to ionophores in both BY4741 and SCY325 genetic backgrounds. (A) Growth of the mutant strains was assessed as described in Fig 1 and in Materials and Methods. (B) Scheme of the pathway of conversion of squalene to ergosterol and phenotypes of the mutants in term of their sensitivity to valinomycin and nigericin, respectively. RD, respiratory deficient.

To address the reproducibility of our results, we performed two experiments. First, we re-constructed the set of deletion mutants in all *ERG* genes, available in the EUROSCARF collection, in the same BY background. We prepared heterozygous diploid (BY4743-derived) strains carrying one standard and one deletion allele of a gene (verified by PCR analysis). The diploids were sporulated and haploid strains carrying the mutant allele were selected. In the case of *ERG24*, we did not obtain any viable Δ*erg24* clone, thus confirming that the gene is essential and that the strain in the EUROSCARF collection probably carries a suppressor mutation or another compensatory genetic change. In the case of *ERG5*, we did not succeed to obtained spores that would exhibit expected sterols profiles and/or PCR fragments and it therefore was not included into the analysis. The identity of the remaining five *erg* mutants (Δ*erg2*, Δ*erg3*, Δ*erg4*, Δ*erg6* and Δ*erg28*) was verified by analysis of cellular sterols (S1 Fig). To verify that in the Δ*erg2* mutant, the peak at 4.27 min (almost identical to the WT’s retention time equal to that of ergosterol) did not correspond to ergosterol, we compared the UV-vis absorption spectra of WT and the Δ*erg2* mutant and we confirmed the absence of this sterol in the mutant (S2 Fig). Three independent clones of each *erg* mutant were subjected to phenotypic tests on media containing nigericin or valinomycin. The mutants exhibited essentially the same phenotypes (S3 Fig) as the EUROSCARF strains shown in the Fig 2.

The second experiment was aimed at investigation of possible effects of interspecific genetic variability on the results. We thus compared the ionophore-affected growth of seven mutants (Δ*erg24*, Δ*erg28*, Δ*erg6*, Δ*erg2*, Δ*erg3*, Δ*erg5*, Δ*erg4*) from the EUROSCARF deletion library (BY4741 genetic background) to the respective deletion strains (with exception of Δ*erg24*) constructed in the W303-derived parental strain SCY325 [38] (Fig 2). Importantly, the sensitivity to ionophores of each *erg* mutant was different from its respective parental strain, but its nature and extent in most cases depended on the genetic background (with the exception of Δ*erg2* and Δ*erg3* which are respiratory-deficient in SCY325 background and thus do not grow on sSG media [41]). This result is in line with different strain-dependent phenotypes already known in mutants in the ergosterol biosynthetic pathway [42]. One of the important differences found between the S288C-derived strains (such as BY4741) and W303-derived (such as SCY325) strains is the mutation *hap1Ty* (in the S288C genome) modulating activity of the transcription factor Upc2p [43]. Upc2p and its paralogue Ecm22p (the latter was identified in this screen as Val^R^), are transcriptional regulators of sterol uptake and bind sterol regulatory elements found in the promoters of most of ergosterol biosynthetic genes [44]. It is therefore plausible that the differences we observed between mutants in BY and W303 are indeed due to some variations in sterol composition.

Thus, the screen uncovered that cellular sterol composition probably affects ionophore action on *S*. *cerevisiae*. In addition, these results, once more, call attention to the big influence that genetic background, with all its known and unknown features, can have on cellular response to a chemical agent or a growth environment.

Interestingly, more than 20% of both Val^R^ and Val^HS^, and almost 15% of Nig^HS^ strains have altered telomere length and/or other defects in maintenance of telomeres (S3 Table). Interestingly, none of the proteins encoded by the genes whose deletion resulted in changes of valinomycin or nigericin sensitivity was shown to be directly associated with chromosomal ends (S4 Table). Instead, they are involved in such diverse biological processes as vacuolar biogenesis and functions (e.g. Vam6p, Vam7p, Pep3p), sterol biosynthesis (e.g. Erg2p), or cell wall biogenesis (e.g. Smi1p). It becomes evident that telomeres are sensitive detectors of cellular stress, yet the architecture of cellular network connecting these genomic loci to other cellular activities is far from being understood [45].

We would like to note that the involvement of some proteins in telomere maintenance indicated by the results of previous systematic screenings must be taken with caution. For example, the Val^R^Nig^R^ mutants Δ*mtc2*, Δ*mtc4*, and Δ*mtc6* were identified in a screen for deletion mutants synthetically lethal with *cdc13-1*. This screen revealed a surprising diversity of genes affecting integrity of chromosomal ends, including genes whose products are involved in mitochondrial functions and ion transport [46]. However, we have found that all these three mutants (Δ*mtc2*, Δ*mtc4*, and Δ*mtc6*) were cold-sensitive, i.e. unable to grow at 15°C (Zeiselova, L., Dzugasova, V., Tomaska, L., data not shown). As the screen of Addinall et al. [46] was based on a search for mutations that affect *cdc13-1* temperature sensitivity, the involvement of *MTC2*, *MTC4* and *MTC6* in telomere maintenance remains questionable.

**Fig 3:**
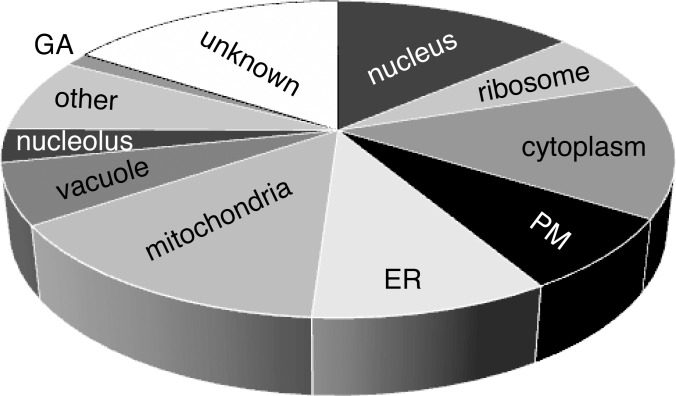
Distribution of localization of the proteins encoded by the genes whose deletion results in altered sensitivity to valinomycin and/or nigericin (see Table 3 for details). Proteins with dual localization are counted twice (once for every localization) to reflect here every occurrence of the proteins in the cell.

Localization of the proteins whose absence leads to an altered sensitivity to ionophores varies to a similar extent as their biological roles (Table 3, Fig 3). As expected, many of them are mitochondrial or vacuolar, but a relatively high number associate with endoplasmic reticulum (ER), cytoplasm, ribosomes, and other cellular structures, emphasizing a complex network of players involved in cellular responses to the ionophores.

**Table 3:** Summary of the numbers of strains categorized based on the localization of the corresponding proteins (see also Fig 3). PM, plasma membrane; ER, endoplasmic reticulum, values in brackets include dual localizations.

Localization	Val^R^	Nig^R^	Val^HS^	Nig^HS^	Val^HS^Nig^HS^	Val^R^Nig^R^	Σ	%
nucleus	9 (13)	1 (2)	2 (2)	1 (1)	2 (2)	0 (0)	15 (20)	11.8 (15.7)
ribosome	9 (9)	0 (0)	0 (0)	0 (0)	0 (0)	0 (0)	9 (9)	7.1 (7.1)
cytoplasm	6 (13)	1 (1)	2 (2)	0 (0)	1 (2)	1 (2)	11 (20)	8.7 (15.7)
PM	4 (7)	0 (0)	1 (2)	2 (2)	0 (0)	0 (0)	7 (11)	5.5 (8.7)
ER	5 (7)	0 (0)	1 (3)	1 (1)	3 (3)	1 (0)	11 (14)	7.9 (11.0)
mitochondria	0 (4)	1 (3)	5 (8)	2 (2)	3 (4)	3 (0)	14 (21)	11.0 (16.5)
vacuole	1 (4)	0 (0)	1 (1)	4 (4)	0 (0)	2 (0)	8 (9)	6.3 (7.1)
nucleolus	3 (4)	0 (0)	0 (0)	0 (0)	1 (1)	0 (0)	4 (5)	3.1 (3.9)
Golgi apparatus	2 (2)	0 (0)	0 (0)	0 (0)	0 (0)	0 (0)	2 (2)	1.6 (1.6)
other	4 (6)	1 (2)	2 (2)	1 (1)	0 (0)	0 (0)	8 (11)	6.3 (8.7)
dual	6	2	2	0	0	0	10	7.9
unknown	12	0	4	2	3	2	23	18.1

### Comparison of the list of the mutants conferring altered sensitivity to nigericin and/or valinomycin with other screens relevant to ion homeostasis

Ionophore sensitivities depend strongly on the growth media used (carbon source used, complex vs synthetic vs semi-synthetic media, solid vs liquid media) (unpublished observation). Very small overlap of our results with the results of screens performed under very different cultivation conditions, using also different concentrations of the ionophores, is therefore not surprising. In a recent fully automatized haploinsufficiency profiling and homozygous deletion profiling assays, testing nearly 1800 biologically active compounds, Hoepfner et al. (2014) [35] identified 160 Nig^HS^, 74 Val^HS^, and 20 Val^HS^Nig^HS^ deletion mutants in the S288C background. Of these, only 5 Nig^HS^ mutants (in *VAM3*, *VAM6*, *VAM7*, *VPS41*, *MON1*), and 2 Val^HS^Nig^HS^ (in *YSP1* and *OSH3*) mutants, were also identified in our screen. Whereas Hoepfner et al. (2014) [35] performed the screen using liquid complex media with glucose (YPD), we used semi-synthetic sSG medium containing glycerol as carbon source. We believe that this is an important difference as both ionophores act on mitochondria and induce formation of respiration-deficient mutants. When glucose is present the result is a mixed population of *rho*^+^ and *rho*^-^/*rho*^0^ cells [21, 23, 26, 27, 32]. Furthermore, Hoepfner et al. (2014) [35] used IC_30_ concentrations of nigericin (11.88 μM) and two “valinomycin derivatives” (3.082 and 4.56 μM). We performed the screening on solid media, searching, besides resistant mutants, for mutants hypersensitive to much lower ionophore concentrations (0.7 μM nigericin, 0.45 μM valinomycin).

Of the 11 mutants identified in our previous “Tn-screen” [27], only 3 corresponding deletion mutants were identified here (S3 Table). Besides possible false negativity and non-identical genetic backgrounds (W303 versus BY4741), some of the discrepancies may be explained by different consequences of a deletion of a gene and its transposon-based mutation (which might partially preserve protein function). Furthermore, the “Tn-screen” may not have been saturated and therefore probably a substantial number of genes were not interrupted by the transposon.

**Fig 4:**
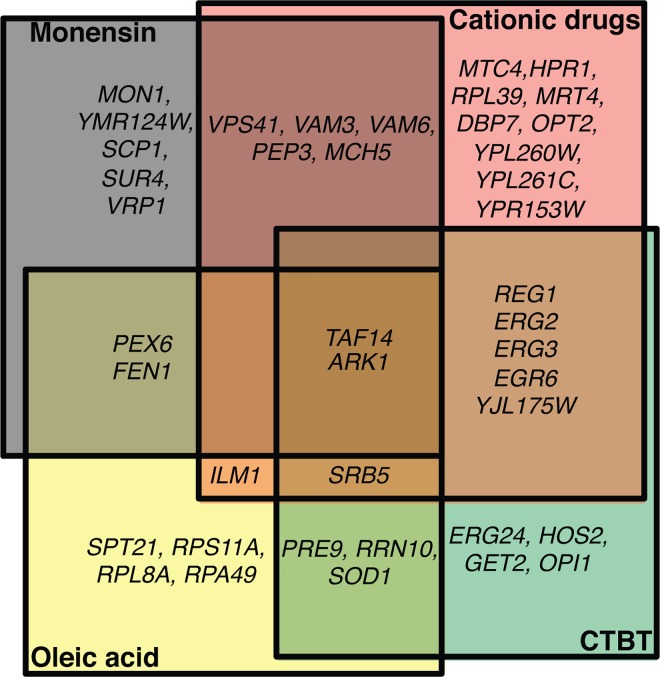
Genes whose absence leads to an altered sensitivity to valinomycin/nigericin as well as at least one of the following compounds: monensin [47], CTBT [48], oleic acid [50], and cationic drugs [52]. See also S5 Table.

To identify phenotypes potentially related to an altered sensitivity to valinomycin and/or nigericin, we compared the list of genes obtained in our study with results of several published screens of the *S*. *cerevisiae* deletion library aimed at identification of mutants exhibiting changed sensitivity to various drugs including valinomycin, nigericin [26, 27], monensin [47], 7-chlorotetrazolo[5,1-c]benzo[1,2,4]triazine (CTBT) [48], palmitoleic acid [49], oleic acid [50], and cationic drugs (spermine, hygromycin B, and tetramethylammonium) [51] (Fig 4, S5 Table).

Since several known nigericin resistant mutants can tolerate also monensin [26], an ionophore catalyzing Na^+^/H^+^ antiport, we were interested also in the co-occurrence of phenotypes related to these two ionophores. We found that the Δ*ymr124w*, Δ*pex6*, and Δ*scp1* mutants were resistant to both nigericin and monensin (40 mg/l). More than half of all Nig^HS^ strains also exhibited hypersensitivity to monensin, including three vacuolar assembly mutants (Δ*mon1*, Δ*vam7*, and Δ*vam3*), Δ*vps41* (lacking a subunit of the homotypic vacuole fusion and vacuole protein sorting (HOPS) complex) and Δ*mch5* (lacking a riboflavin transporter). All of these mutants were also identified in a screen for mutations leading to defects in mitophagy [51]. Of the 63 monensin hypersensitive mutants identified by [47], only 11 were also on our list: Δ*vam6*, Δ*vps41*, Δ*vam7*, Δ*vam3*, Δ*akr1*, and Δ*mon1* from the Nig^HS^ group, Δ*pep3*, Δ*sur4* (Δ*elo3*), and Δ*vrp1* from the Val^HS^ group and Δ*taf14* and Δ*fen1* (Δ*elo2*) from Val^HS^Nig^HS^ mutants (Fig 4, S5 Table). The discrepancies between the results of the two screens are partially due to the different cultivation conditions (complete SD [47] versus sSG [26]) and possibly also in monensin concentrations: 35 mg/l [47] versus 40 mg/l [26] in our study.

Almost 19% (24) of the mutants exhibiting altered sensitivity to valinomycin and/or nigericin had been identified as sensitive to cationic drugs (spermine, hygromycin B, and tetramethylammonium) and several also exhibited growth defects at a limiting potassium concentration [52] (Fig 4, S5 Table). Several mutants identified in our “Tn-screen” were also shown to exhibit sensitivity to oleic acid [50] and this phenotype had also been described in 12 of the now identified deletion mutants (Fig 4, S5 Table). Furthermore, 18 of the mutants are also hypersensitive to palmitoleic acid [49, 50]. Finally, 21 mutant strains with altered sensitivity to ionophores were also hypersensitive to the chemosensitizing agent 7-chlorotetrazolo[5,1-c]benzo[1,2,4]triazine (CTBT) known to induce oxidative stress and thus enhance the effect of several antifungals [48] (S5 Table).

Only two deletion mutants, Δ*akr1* and Δ*taf14*, exhibited hypersensitivity to as many as five of the mentioned compounds (nigericin and/or valinomycin, monensin, CTBT, oleic acid, and cationic drugs), *akr1*^-^ also tolerated increased valinomycin concentration. *AKR1* encodes vacuolar protein-cysteine S-palmitoleyltransferase involved in palmitoylation of a wide repertoire of proteins and thus affecting a wide variety of cellular functions. Similarly, the deletion of *TAF14*, encoding a general RNA polymerase II transcription factor, results in global changes in cellular proteome and it is impossible to pinpoint the mechanisms leading to the described hypersensitivities. According to *FunSpec* analysis, the other mutants with overlapping phenotypes were enriched for genes whose products are involved in lipid (sterol and fatty acids (FA)) biosynthesis and vacuolar biogenesis (data not shown).

### Nigericin induces rapid changes in the vacuolar morphology and intracellular pH

**Fig 5:**
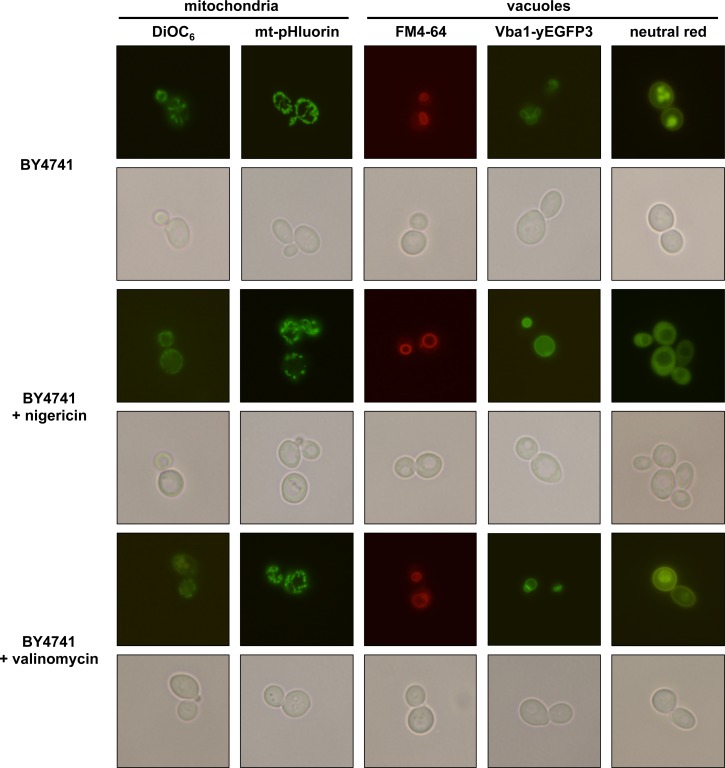
Organelle morphology in yeast cells treated with ionophores. The wild-type strain (BY4741) and the corresponding transformants were cultivated in sSG medium. After addition of nigericin (15 mg/l) or valinomycin (15 mg/l) the cells were observed by fluorescence microscopy. Mitochondria were visualized using DiOC_6_ or mt-pHluorin (expressed from a plasmid) and vacuoles were stained with FM4-64, neutral red or Vba1-yEGFP3 (expressed from the plasmid pDF01).

To investigate the effect of nigericin and valinomycin on mitochondria and vacuoles, we cultivated the wild-type cells in the sSG medium and, immediately after addition of the ionophore, the organelles were observed by fluorescence microscopy (Fig 5, S6 Table). We found that the cells did not exhibit significant changes of the mitochondrial network as shown by using both DiOC_6_, which stains depending on mitochondrial membrane potential [53], and mt-pHluorin, a pH-sensitive GFP targeted into mitochondria [39]. However, we observed profound alterations of the vacuolar morphology and intracellular pH in nigericin-treated cells. This ionophore induces rapid formation of a large central vacuole as revealed by FM4-64, which under pulse-chase conditions stains vacuolar membranes [54]. Similar results were observed in transformants expressing Vba1-yEGFP3, a vacuolar permease for basic amino acids tagged with a GFP derivative. Moreover, the staining with neutral red, a pH indicator for acidic subcellular compartments, revealed that nigericin treatment led to increased vacuolar pH and cytosol acidification. In contrast, valinomycin induces only mild changes in intracellular pH. While the increased vacuolar pH and cytosol acidification occur in about 98% of nigericin-treated cells, similar cytological phenotype was present in less than 12% of cells treated with valinomycin (S6 Table).

## Discussion

The *Saccharomyces* Genome Deletion Project [55, 56] has enabled new approaches to uncovering phenotypes related to the removal of any of the nonessential genes. We are aware that the screening procedure, as well as the library itself, poses some limitations. Due to the large-scale process, many strains being cultivated, diluted, and dropped in parallel, the slowly growing mutants are plated in a smaller density than the others. Even though their growth under considered conditions is compared to their growth on the ‘positive control plate’ (where they were plated at the same density), the results are sometime ambiguous. Our unpublished observations suggest that the yeast sensitivity to ionophores negatively correlates with the quantity of cells plated on media with the same concentration of the drug (data not shown). This would be in accordance with, for instance, observed action of monensin on bacteria [57, 58], during which the ratio of ionophore to cell material appeared as more important for physiological effects than the absolute ionophore concentration.

Even plating the mutants always in quadruplicates does not eliminate problems with growth evaluation. Three fourths of the drops on the same plate should have the same phenotype to allow us considering the mutant as ‘candidate’. However, in general, the observed drops differed considerably: some including many individual small colonies, some others only several bigger colonies. After retesting of some of these mutants, we decided to consider these ‘colonies providing’ strains as ‘false positives’ and did not include them among the candidate strains.

Our observations lead us to a conclusion worth considering also when evaluating different genetic screens as well as some other experiments. Cultures of many mutants are genetically or physiologically heterogeneous to the extent that a part of their cells is able to form colonies on ionophore-containing media. These phenomena may remind bacterial adaptive mutations and persistence, respectively. Mutation rates increase in response to stress, so beneficial mutations can be produced (for review, see [59]). Alternatively, bacteria can enter a transient physiological state, persistence, in which antibiotics do not affect them. Contrary to earlier assumption, persisters do not occur stochastically prior to antibiotic treatment, but the majority of them is formed in response to the treatment [60]. We can thus speculate that some yeast mutations might increase either the mutation rate, or the likelihood of entering a state similar to bacterial persistence.

In yeast stress conditions activate responses that include stress neutralization, pausing of the cell cycle, alternations in translation, and damage repair [61]. An important component of stress responses is the control of RNA metabolism, often involving a decrease in general translation and preferential translation of mRNAs functioning in stress responses (for review, see [62]). However, none of the mutants we identified as hypersensitive to at least one ionophore is impaired in a component of the translational machinery, in contrast to 21% of the Val^R^ mutants.

A related practical problem with the screening procedure and results evaluation is also the variability in phenotypes throughout retesting. This phenomenon has been encountered also by other authors and is presumably caused by some changes in cellular physiology and/or subtle changes in growth conditions (see for instance [50]). It is not typical for mutants only, as also the sensitivity of all WT strains we tested varied slightly from experiment to experiment.

Finally, the collection of deletion mutants is supposed to be isogenic for all the ‘background’ genes. In fact, it was shown that most of the knockout strains acquired additional mutations selected to counteract the effect(s) of the original genetic defect [63] or background mutations contribute to the observed phenotype [35]. For example, the strains containing deletions of ORFs located between *YOR097C* and *YOR192C*, including *MDM32*, had an additional mutation in the *MSH3* mismatch repair gene [64]. The authors observed elevated rates of microsatellite instability caused by this *msh3* mutation. Moreover, in some of the mutants, the deletion cassette is integrated in other locus than stated. As mistakes are being discovered, newly prepared and verified mutants are added to the library. In many cases, the candidate mutations are tested in the opposite mating type and/or in a different genetic background and differences in observed phenotypes are not unusual. Even the monensin sensitivity of some mutants was shown to be modulated by genetic background [47]. We observed some discrepancies with our previous results: the Δ*mdm32* strain from the library was not Nig^R^ (yet surprisingly was still Val^HS^) and the Δ*erg24* was Val^R^Nig^R^ even though it did not display this phenotype previously [26]. Furthermore, Δ*trk1* and Δ*trk2*, which lack the plasma membrane K^+^ transporter and are slightly hypersensitive to both ionophores (Petrezselyova, S., Sychrova, H., personal communication) [0.5 mg/l nigericin, 3 mg/l valinomycin], grew as the wild type under our conditions.

In spite of these limitations, the deletion library provides powerful means for genome-wide analyses and identification of genes potentially involved in a given biological process. What conclusions can be drawn from our screen for mutants with an altered sensitivity to valinomycin and nigericin? One would expect that the majority of mutants could not maintain appropriate ion concentrations in cellular compartments, have a defect in a process responsive to disturbances of certain (electro) chemical gradients, or have an altered membrane lipid composition. Indeed, we identified strains deficient in an ion transporter, sensitive to hyperosmotic stress or to metal ions, or impaired in lipid synthesis in our screens.

Interestingly, the majority of the strains we obtained when looking for altered sensitivity to valinomycin and/or nigericin are mutated in genes without any apparent connection to ion homeostasis or membrane permeability. 18% of the Val^R^ mutants have disturbed translation or ribosome biogenesis, while other groups do not include ribosomal mutants. This potential involvement of ribosomes in valinomycin action on yeast reminds experiments on rabbit reticulocytes. In this model system, valinomycin reversibly inhibits proteosynthesis at the level of elongation, at concentrations where its action as an ionophore cannot be detected [65]. Incubation of rabbit reticulocytes with 10^−5^ mol/dm^3^ (11 mg/l) valinomycin, sufficient for K^+^ exchange disturbance, leads to the breakdown of ribosomes, thus an irreversible inhibition of their function [66]. If a similar effect, albeit not yet described, occurs in yeast, then translational defects could likely lead to hypersensitivity to this compound, as destabilized ribosomes could be more prone to additional damage. However, we did not find this type of mutants among the Val^HS^. One possible explanation of the mechanism(s) leading to the resistance to valinomycin in mutants missing a component of the cytoplasmic ribosomal small subunit can be as follows: the ionophore can negatively modulate translation by specifically binding to the small ribosomal subunit, and thus removing a component essential for ionophore binding hampers this inhibition. This would suggest that the action of valinomycin on the cell could be on two parallel levels–at ribosomes and at membranes–and removal of one of the effects would be sufficient to increase cell survival. Alternatively, the aberrant ribosomes could induce secondary changes in the cell, which somehow compensate the otherwise detrimental effect of the ionophore. These changes could be too complex to theoretically deduce them from the character of the mutation.

When speculating about possible involvement of ribosomal proteins or even RNAs, as well as of open reading frames (ORFs) with so far unknown function in modulation of cellular response to valinomycin and nigericin another possibility is worth considering. Ribosomes are also direct targets for small regulatory non-coding RNAs (ncRNAs) [67]. Mutations in some ‘dubious genes’ or uncharacterized ORFs (about 20.5% in our screen), but even in some already partially characterized genes, might affect formation of these regulatory RNA molecules. On the other hand, mutations in a ribosomal component might prevent interaction of regulatory RNA molecules with the ribosomal target site. So far, only one ribosome-binding ncRNA has been characterized. This 18-nt-long fragment derived from the *TRM10* locus inhibits global protein biosynthesis and this attenuation is crucial for adaption under hyperosmotic stress [67].

Several defects in lipid biosynthesis led either to ionophore resistance or hypersensitivity indicating the role of membranes’ composition on ionophore tolerance (Fig 2). Although the effect of deletion of individual genes on drug sensitivity was strain-specific, the results indicate that the changes in the composition of cellular membranes affect the cell's response to ionophores. The lipid metabolism mutants exhibiting resistance to ionophores include the Δ*erg24* lacking the sterol C-14 reductase and accumulating mainly aberrant sterol ignosterol (ergosta-8,14-dienol), Δ*erg6* lacking the sterol C-24 methyltransferase converting zymosterol to fecosterol, Δ*erg2* without the sterol C-8 isomerase, and Δ*ecm22* missing a sterol regulatory element binding protein [68]. The absence of a negative transcriptional regulator of phospholipid biosynthetic genes, Opi1p, [69], also leads to increased valinomycin tolerance. Based on published data, we cannot say if there is one specific trait, common for all these mutants resulting in the resistance to valinomycin. *ERG24*, *ERG2*, and *OPI1* deletions can be suppressed by the deletion(s) of *SUR4* or *FEN1* [70, 71], encoding elongases, synthesizing very long chain 20-26-carbon FA that are components of another class of lipids, sphingolipids. In contrast, Δ*erg6* and Δ*sur4* are synthetically lethal [72]. Interestingly, absence of Sur4p and Fen1p elongases leads in our screen to Val^HS^ phenotype. An unambiguous relation between membrane composition and ionophore sensitivity was observed also in other systems and with other ionophores. For instance, in bacteria, a single mutation in the FA desaturase system resulting in an increase in the saturated/unsaturated FA ratio of membrane phospholipids affects dramatically their protonophore sensitivity as well as their bioenergetic phenotype (for review, see [73]).

Mitochondria are dependent on phospholipid supply from the ER via lipid exchange at membrane contact sites. At least one of two types of such contact sites is essential; ERMES (ER-mitochondria encounter structure) composed of Mmm1p, Mdm10, Mdm12 and Mdm34 [74] or vCLAMP (vacuole and mitochondria patch) containing Vam6 and Vps39 [75]. Deletion of *VAM6* (as well as of *VAM3*, *VAM7*, *VPS41*, *MON1* or *OPT2* and, under different conditions, of other 48 vacuolar-related genes [35]) leads to nigericin hypersensitivity.

It is noteworthy that 9 ionophore-hypersensitive and 15 resistant mutants (S5 Table) also exhibit sensitivity to cationic drugs. Any mutant displaying reduced tolerance concurrently to diverse toxic cations was presumed to be affected in the electrochemical gradient across its plasma membrane [52].

Ionophore resistance of the identified mutants was not related to a generally improved tolerance to toxic compounds, moreover it was often accompanied by an increased vulnerability to some toxins (described by other authors, see Saccharomyces Genome Database). Similarly, ionophore hypersensitivity is not generally linked to a pronounced susceptibility to exogenous substances.

The Nig^R^ mutants include Δ*pex6* and Δ*sod1* with increased ROS levels and decreased oxidative stress resistance and lifespan due to lack of the peroxisomal AAA-ATPase Pex6p [76]. Although the *FunSpec* analysis indicated “replicative cell aging” as an over-represented category in our screen for Nig^R^ mutants, we cannot conclude that nigericin tolerance somehow relates to this process. The relation is more likely due to the ROS production and/or oxidative stress, but the nature of the influence is still impossible to presume.

Mutants in *MDM31* or *MDM36* (as well as *MDM32* not obtained in this screen) have abnormal mitochondrial morphology [28]. Nigericin can alleviate some of the morphological defects (as observed in Δ*mdm31*, Δ*mdm32* [27], and several UV-induced mutants [26]). The examination of Δ*mdm36* cells with and without nigericin did not reveal any noticeable influence of the ionophore on mitochondrial morphology.

Among the 21 Val^HS^ mutants there are strains missing FA elongases, Fen1p or Sur4p. Both are involved in sphingolipid biosynthesis, Fen1p by providing FA up to 24 carbons in length, Sur4p the very long chain 20-26-carbon FA from C18-CoA primers [77, 78]. Fen1p has regulatory effects on 1,3-β-glucan synthase, vacuolar ATPase, and the secretory pathway [79–81]. Sur4p is involved in regulation of sphingolipid synthesis; [77, 78] in its absence, ceramide accumulates [82]. Both mutants, Δ*fen1* and x*sur4*, are resistant to fenpropimorph, an inhibitor of yeast sterol reductases [83].

The effect of ionophores on vacuolar functions is apparent from their morphological changes, increased vacuolar pH and cytosol acidification observed in the wild-type cells immediately after the addition of nigericin. In addition, mutants defective in vacuole-related functions (i.e. Δ*vam3*, Δ*vam6*, Δ*vam7*, Δ*vps41*, Δ*mon1* identified in our screens as well as almost 50 vacuolar mutants identified in [35] are Nig^HS^; Δ*opt2* is Val^HS^Nig^HS^; Δ*pep3* is Val^HS^; Δ*ape3*, Δ*atg15* and Δ*yml018c* are Val^R^) showed altered sensitivity to ionophores.

In summary, although the initial studies [21, 23] on the effect of valinomycin and nigericin on yeast cells highlighted mitochondria as one of their principal cellular targets, cumulative evidence indicates that the cellular response to these ionophores is very complex. Our results emphasize that the treatment of cells with ionophores perturbing ion homeostasis affects an intricate network of proteins connecting mitochondria, vacuoles and other membrane compartments. Identification of these proteins presented in this study is the first step toward understanding the molecular details of this network.

## Supporting Information

S1 Fig Sterol profiles of wild type strain (blue), viable *erg* mutants in W303 background (green) and *erg* mutants in BY background (red). Sterols were isolated and HPLC analyzed as described in Material and Methods. Ergosterol and its precursors elute within time interval 2.8 to 6.0 minutes. Red arrow indicates retention time for ergosterol (4.27 min).
(PDF)

S2 Fig The UV-vis absorbtion spectra of ergosterol peak in wild type strain and of ergosterol precursor peak with retention time of 4.27 min in Δ*erg2* mutant. Spectral analysis was performed using DAD detector.
(PDF)

S3 Fig Sensitivity to ionophores in independent *erg* mutant clones constructed in the BY background. Sensitivity to ionophores in independent *erg* mutant clones constructed in the BY background. The growth of the mutant strains was assessed as described in Fig 1 and Fig 2 in the main text. The table summarizes the phenotypes of all erg mutants tested in this study. RD, respiration-deficient strain; S1-S3, 3 independent spores with the corresponding genotype; E, the mutant strains from the EUROSCARF collection; SCY, the mutant strains made in the SCY325 background; N.D., not done; *, see Fig 2 in the main text.
(PDF)

S1 Table Concentrations of used drugs. (PDF)

S2 Table Sequences of primers used for construction and verification of *erg* deletion mutants in BY background. (PDF)

S3 Table List of mutants exhibiting an altered sensitivity to valinomycin and/or nigericin. (XLS)

S4 Table List of genes involved in both mediating the effect of valinomycin and/or nigericin and maintenance of nuclear telomeres. (PDF)

S5 Table List of genes whose absence leads to an altered sensitivity to ionophores as well as other selected drugs. (PDF)

S6 Table Staining of the control (wild-type) and ionophore-treated yeast cells using neutral red. (PDF)
