# Supplementary material for: Identification of Yeast Mutants Exhibiting Altered Sensitivity to Valinomycin and Nigericin Demonstrate Pleiotropic Effects of Ionophores on Cellular Processes
Source: PLoS One. 2016 Oct 6;11(10):e0164175. doi: 10.1371/journal.pone.0164175 (PMC5053447; doi:10.1371/journal.pone.0164175)
Supplement: S1 Fig — Sterols were isolated and HPLC analyzed as described in Material and Methods. Ergosterol and its precursors elute within time interval 2.8 to 6.0 minutes. Red arrow indicates retention time for ergosterol (4.27 min). (PDF) [file pone.0164175.s001.pdf]

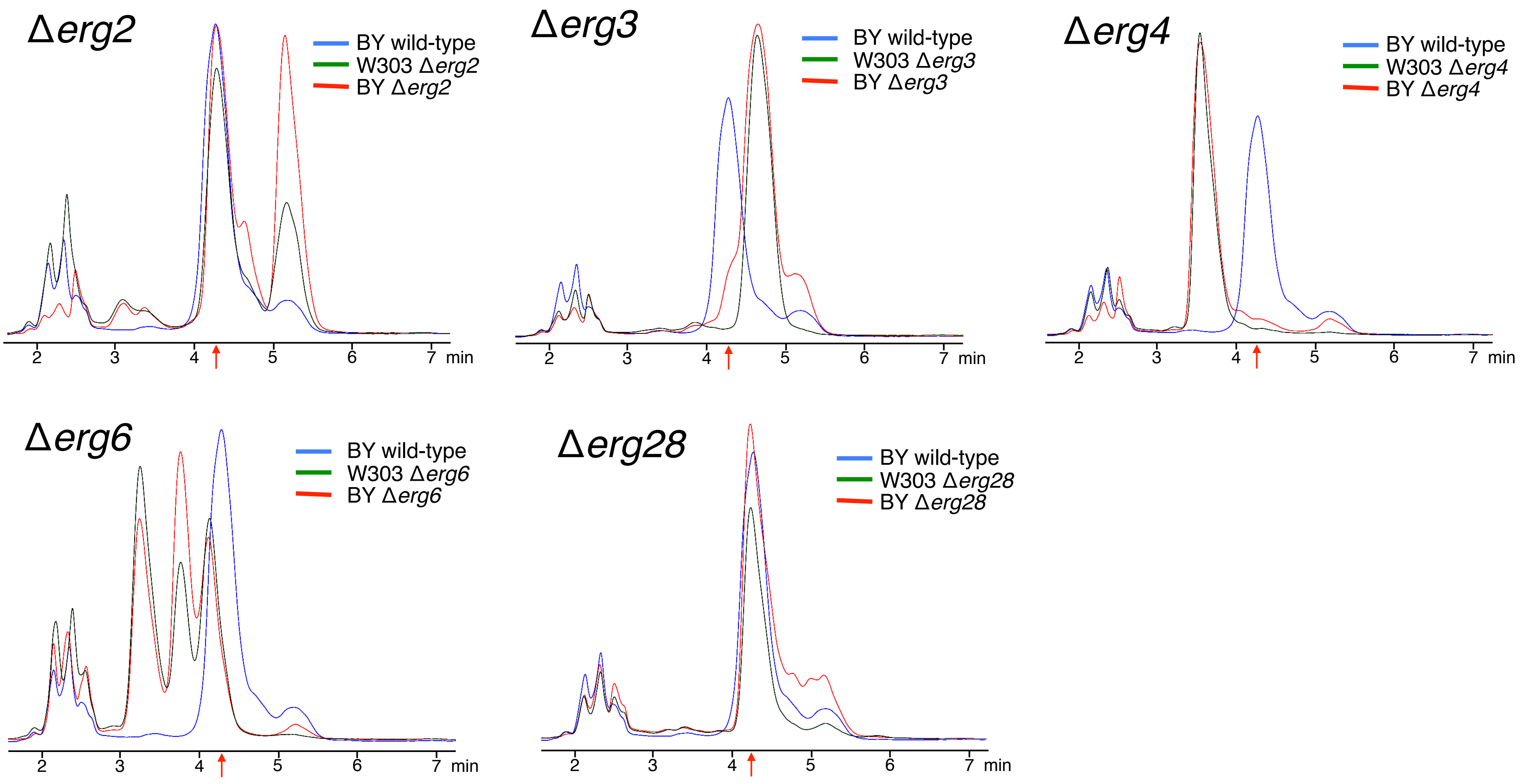

S1 Figure. Sterol profiles of wild type strain (blue), viable *erg* mutants in W303 background (green) and *erg* mutants in BY background (red). Sterols were isolated and HPLC analyzed as described in Material and Methods. Ergosterol and its precursors elute within time interval 2.8 to 6.0 minutes. Red arrow indicates retention time for ergosterol (4.27 min).
