# Supplementary material for: Identification of Yeast Mutants Exhibiting Altered Sensitivity to Valinomycin and Nigericin Demonstrate Pleiotropic Effects of Ionophores on Cellular Processes
Source: PLoS One. 2016 Oct 6;11(10):e0164175. doi: 10.1371/journal.pone.0164175 (PMC5053447; doi:10.1371/journal.pone.0164175)
Supplement: S2 Fig — Spectral analysis was performed using DAD detector. (PDF) [file pone.0164175.s002.pdf]

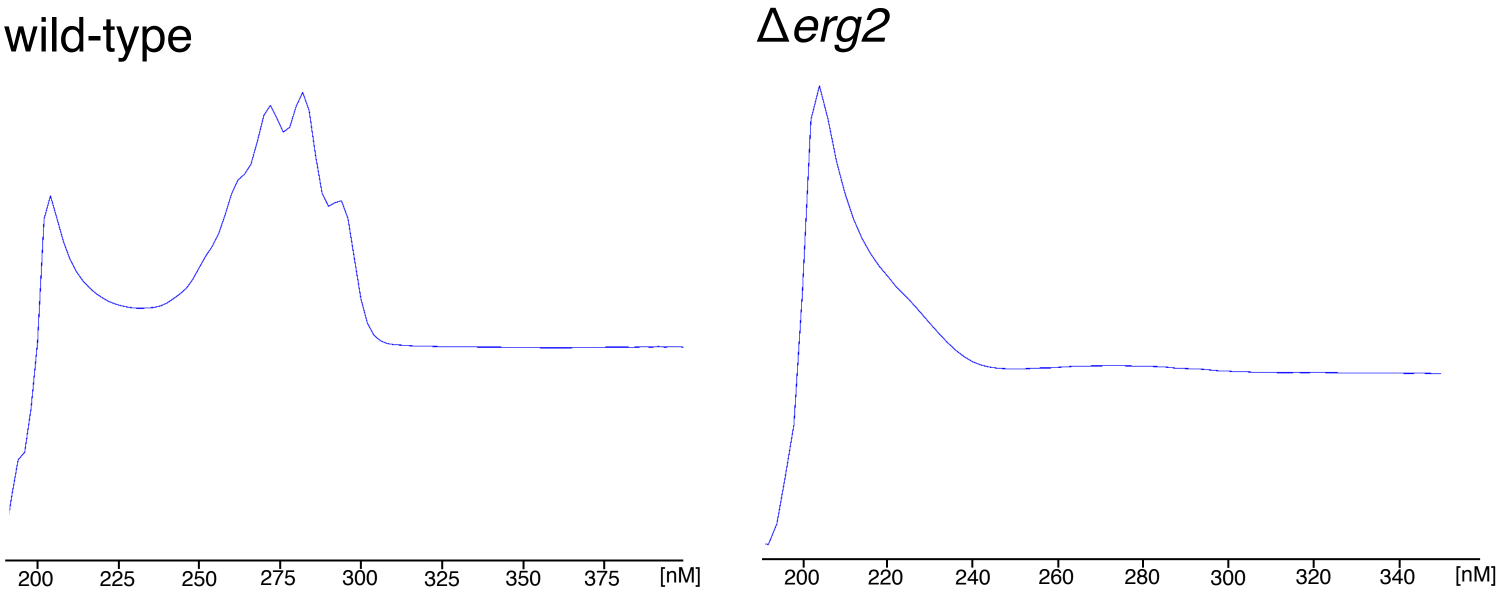

S2 Figure. The UV-vis absorbtion spectra of ergosterol peak in wild type strain and of ergosterol precursor peak with retention time of 4.27 min in Δ*erg2* mutant. Spectral analysis was performed using DAD detector.
