# Supplementary material for: Identification of Yeast Mutants Exhibiting Altered Sensitivity to Valinomycin and Nigericin Demonstrate Pleiotropic Effects of Ionophores on Cellular Processes
Source: PLoS One. 2016 Oct 6;11(10):e0164175. doi: 10.1371/journal.pone.0164175 (PMC5053447; doi:10.1371/journal.pone.0164175)
Supplement: S3 Fig — Sensitivity to ionophores in independent erg mutant clones constructed in the BY background. The growth of the mutant strains was assessed as described in Fig 1 and Fig 2 in the main text. The table summarizes the phenotypes of all erg mutants tested in this study. RD, respiration-deficient strain; S1-S3, 3 independent spores with the corresponding genotype; E, the mutant strains from the EUROSCARF collection; SCY, the mutant strains made in the SCY325 background; N.D., not done; *, see Fig 2 in the main text. (PDF) [file pone.0164175.s003.pdf]

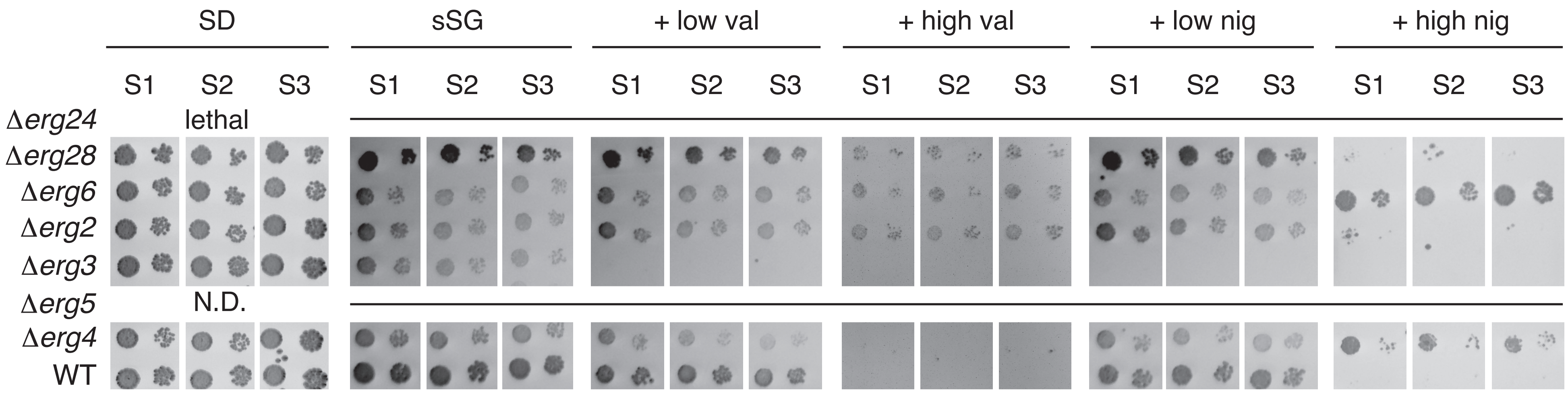


| | S1 | S2 | S3 | E* | SCY* |
|---|---|---|---|---|---|
| *Δerg24* | lethal | lethal | lethal | Val$^{R}$Nig$^{R}$ | lethal |
| *Δerg28* | Val$^{R}$ | Val$^{R}$ | Val$^{R}$ | Nig$^{R}$ | Val$^{R}$ |
| *Δerg6* | Val$^{R}$Nig$^{R}$ | Val$^{R}$Nig$^{R}$ | Val$^{R}$Nig$^{R}$ | Val$^{R}$Nig$^{R}$ | Val$^{R}$ |
| *Δerg2* | Val$^{R}$ | Val$^{R}$ | Val$^{R}$ | Val$^{R}$ | RD |
| *Δerg3* | Val$^{HS}$Nig$^{HS}$ | Val$^{HS}$Nig$^{HS}$ | Val$^{HS}$Nig$^{HS}$ | Val$^{HS}$Nig$^{HS}$ | RD |
| *Δerg5* | N.D. | N.D. | N.D. | Val$^{R}$ | Val$^{R}$ |
| *Δerg4* | Nig$^{R}$ | Nig$^{R}$ | Nig$^{R}$ | Nig$^{R}$ | Val$^{HS}$ |

**S3 Figure**. Sensitivity to ionophores in independent *erg* mutant clones constructed in the BY background. The growth of the mutant strains was assessed as described in Fig 1 and Fig 2 in the main text. The table summarizes the phenotypes of all *erg* mutants tested in this study. RD, respiration-deficient strain; S1-S3, 3 independent spores with the corresponding genotype; E, the mutant strains from the EUROSCARF collection; SCY, the mutant strains made in the SCY325 background; N.D., not done; *, see Fig 2 in the main text.
