# Supplementary material for: Identification of Yeast Mutants Exhibiting Altered Sensitivity to Valinomycin and Nigericin Demonstrate Pleiotropic Effects of Ionophores on Cellular Processes
Source: PLoS One. 2016 Oct 6;11(10):e0164175. doi: 10.1371/journal.pone.0164175 (PMC5053447; doi:10.1371/journal.pone.0164175)
Supplement: S1 Table — (PDF) [file pone.0164175.s004.pdf]

S1 Table.
Concentrations of used drugs

Drug	Stock solution (concentration in g/l, solvent)	Concentration in the medium (in mg/l)	Medium
valinomycin	10, DMSO	*see results*	sSG
nigericin	10, methanol	*see results*	sSG
monensin	10, methanol	*see results*	sSG
oligomycin	1, methanol	1	YPG
antimycin A	2, ethanol	2	YPG
fluconazole	5, H_2O	150	YPD
ethidium bromide	10, H_2O	1	YPG
cycloheximide	10, ethanol	1	YPD
G418 sulphate	100, H_2O	200	YPD
