# Supplementary material for: Identification of Yeast Mutants Exhibiting Altered Sensitivity to Valinomycin and Nigericin Demonstrate Pleiotropic Effects of Ionophores on Cellular Processes
Source: PLoS One. 2016 Oct 6;11(10):e0164175. doi: 10.1371/journal.pone.0164175 (PMC5053447; doi:10.1371/journal.pone.0164175)
Supplement: S2 Table — (PDF) [file pone.0164175.s005.pdf]

S2 Table. Sequences of primers used for construction and verification of *erg* deletion mutants in BY background

Primer name	DNA sequence (5'->3')*
frwERG2kanMX	AGAAGCGGTAACGTTTGACACTGGGTTCAGATCTCTCTTGTCGCTCAATCAAACTAAGACTAGCCCAGACCATTATAGCCCGTACGCTGCAGGTCGAC
revERG2kanMX	ATAATAATAGTAATAGTAATAATAATAATAGTTATATTATAATGGACTACCGCATGACTGATTTCGTGAGGTCGGGCAGCATCGATGAATTCGAGCTCG
frwERG2chck	ATGCAATGCACATTCCTGCC
revERG2chck	TCATGTCCACCAAAAGACGC
frwERG3kanMX	TTGAAGTGGTTGCAGAGGAGGTCAGTTTGTTGCATTTGTAAAAAAAGATAATAAGAAAAATATTCGTCTAGATTTGAGATCGTACGCTGCAGGTCGAC
revERG3kanMX	ATATATAAACGTTATTCGTTGTGTGAAAGTTCTTGAACGTGAAAGAAAGAAAAAAGATGAGACAAACAAGGCAACCGTATATCGATGAATTCGAGCTCG
frwERG3chck	TTCAGGCTCGTATAAGTGGC
revERG3chck	TTAACACTTGCGCTTCACCG
frwERG4kanMX	GTAATAACGTAGGAATGTAGTTCAGTTATGTAGATAGGCAGATACGGATATTTACGTAGTGTACATAGATTAGCATCGCTCGTACGCTGCAGGTCGAC
revERG4kanMX	CTTTTGTCGCGTAAATACATCAATACTTTTATATACAAACTGTAAAATAAGTTAATGAAGTGGATAGAAAAAGAAAATAAATCGATGAATTCGAGCTCG
frwERG4chck	CTCCATTTAGGGTATGCGTC
revERG4chck	CTTGAGTGCTGAGAACATCG
frwERG5kanMX	ATTGTTTCCTTAATTTTTATCACAATAAACAAAACATCACATTTTGCTATTCCAATAGACAATAAATACCTTTTAACAAACGTACGCTGCAGGTCGAC
revERG5kanMX	GAAAATAAAAGTATTCAAAACGCCAACCCTTAATGAAGTAAATATGATTTATTGTCTGGACAAAGTTCTGTTTTTCCCCAATCGATGAATTCGAGCTCG
frwERG5chck	GAAGAGAGCTCATGTTTCGG
revERG5chck	TCGCCTTCACGGAACTTAGT
frwERG6kanMX	TTATCTGTTTTACTTTCGATTTAAGTTTTACATAATTTAAAAAAACAAGAATAAAATAATAATATAGTAGGCAGCATAAGCGTACGCTGCAGGTCGAC
revERG6kanMX	CGTGCTATCTTTTTATCTGCATATATAGGAAAATAGGTATATATCGTGCGCTTTATTTGAATCTTATTGATCTAGTGAATATCGATGAATTCGAGCTCG
frwERG6chck	CCTCCAATACTTGCTGTTGC
revERG6chck	TGTAGGGGAGCAGTCAATAC

frwERG24kanMX	TTTTCTTGCGCAATTGCTTATCAGATAGACCTTGTAAACAGCATAGGAGTAAAGACAAATTCGGTGTAGAGAATAAAAGGCGTACGCTGCAGGTCGAC
revERG24kanMX	TTCTTCCTCATACTCACCCAATACATAACATGTATACACACATACATAGATAATGAAGAAAATAGCAGTGGTAGAAAAACATCGATGAATTCGAGCTCG
frwERG24chck	GCATTGTGTGAAGGTTGTGC
revERG24chck	TTCCGATTTTGTCGCAAGGG
frwERG28kanMX	GACTAATTTCAGGTTTTTATATTCTTGACACTAGCTAGACCATAGTATCGAAGGATTCAAATACACTAAAGTATCAGATACGTACGCTGCAGGTCGAC
revERG28kanMX	AAAGGTTGCTATAGGCATTATATAAATAAATCATCTCTATATAATTTTTTTTACAGGATATGCTTGCCCTTACATCAGAAATCGATGAATTCGAGCTCG
frwERG28chck	CCGGACTTGCCCATGATTAA
revERG28chck	CGAGATCACGTGCACAGAAA
kanB	CTGCAGCGAGGAGCCGTAAT
kanC	TGATTTTGATGACGAGCGTAAT

*underlined - sequences homologous to *kanMX4* cassette
