# Supplementary material for: Identification of Yeast Mutants Exhibiting Altered Sensitivity to Valinomycin and Nigericin Demonstrate Pleiotropic Effects of Ionophores on Cellular Processes
Source: PLoS One. 2016 Oct 6;11(10):e0164175. doi: 10.1371/journal.pone.0164175 (PMC5053447; doi:10.1371/journal.pone.0164175)
Supplement: S4 Table — (PDF) [file pone.0164175.s007.pdf]

S4 Table.

List of genes involved in both mediating the effect of valinomycin and/or nigericin and maintenance of nuclear telomeres.

Gene	Gene function	Val/Nig	Telomeres	*cdc13-1*#
HPR1	DNA/RNA transactions	Val^R	L*	-
SRB5	RNA transcription	Val^R	S*	-
SUR4	Fatty acid elongase	Val^{HS}	S*	+
SMI1	Regulation of cell wall biogenesis	Val^{HS}	S*	-
OPI1	Transcriptional regulator	Val^R	S*	-
MRT4	Ribosome biogenesis and RNA turnover	Val^R	S*	+
APE3	Vacuolar aminopeptidase Y	Val^R	S*	-
HCM1	Forkhead transcription factor	Val^R	L*	+
PEP3	Vacuolar biogenesis	Val^{HS}	S*	-
CTF8	Mitotic sister chromatid cohesion	Val^R	S**	-
RSA1	Ribosome biogenesis	Val^R	L**	-
RPS4A	Protein component of the small ribosomal subunit	Val^R	L**	-
RPS16A	Protein component of the small ribosomal subunit	Val^R	L**	-
RPS16B	Protein component of the small ribosomal subunit	Val^R	L**	-
RPS27B	Protein component of the small ribosomal subunit	Val^R	L**	-
SRB8	RNA transcription	Val^R	L**	-
ERG2	C-8 sterol isomerase	Val^R	S**	+
SPT21	Protein with a role in transcriptional silencing	Val^{HS}	S**	-
VAM6	Vacuolar biogenesis	Nig^{HS}	S***	+
VAM7	Vacuolar biogenesis	Nig^{HS}	S***	+
FEN1	Fatty acid elongase	Val^{HS}	N	+
ERG6	Δ(24)-sterol C-methyltransferase	$Val+Nig^R$	N	+
ILM1	Peroxisomal protein involved in regulation fatty acid biosynthesis	$Val+Nig^{HS}$	N	+
MGR2	Subunit of the TIM23 translocase complex	Val^{HS}	N	+
MTC2	Maintenance of telomere capping	$Val+Nig^R$	N	+
MTC4	Maintenance of telomere capping	$Val+Nig^R$	N	+
MTC6	Maintenance of telomere capping	$Val+Nig^R$	N	+
PHO5	Acid phosphatase	Val^R	N	+
DBP7	Translation and ribosome biogenesis	Val^R	N	+
PRE9	Subunit of the 20S proteasome	Val^R	N	+

*[1]; **[2], ***www.yeastgenome.org; #[3]; N, normal length; S, short telomeres; L, long telomeres

References:

[1] Askree SH, Tzfati Y, Smolikov S, Gurevich R, Hawk J, Coker C et al. (2004). A genome-wide screen for Saccharomyces cerevisiae deletion mutants that affect telomere length. Proc Natl Acad Sci USA 101: 8658-8663.
[2] Gatbonton T, Imbesi M, Nelson M, Akey JM, Ruderfer DM, Kruglyak L et al. (2006) PLoS Genet 2: e35.
[3] Addinall SG, Downey M, Yu M, Zubko MK, Dewar J, Leake A et al. (2008) A genomewide suppressor and enhancer analysis of cdc13-1 reveals varied cellular processes influencing telomere capping in Saccharomyces cerevisiae. Genetics 180: 2251-2266.
