# Supplementary material for: Identification of Yeast Mutants Exhibiting Altered Sensitivity to Valinomycin and Nigericin Demonstrate Pleiotropic Effects of Ionophores on Cellular Processes
Source: PLoS One. 2016 Oct 6;11(10):e0164175. doi: 10.1371/journal.pone.0164175 (PMC5053447; doi:10.1371/journal.pone.0164175)
Supplement: S5 Table — (PDF) [file pone.0164175.s008.pdf]

S5 Table. List of genes whose absence leads to an altered sensitivity to ionophores as well as other selected drugs

ORF	GENE	PREDICTED FUNCTION OF THE PROTEIN	VAL [1]	NIG [1]	MON [1]	MON [3]	CTBT [4]	PA [5]	OA [6]	KD [7]	HRCD [8]
YDR264C	*AKR1*	Protein-cysteine S-palmitoleyltransferase	R	HS		HS	HS		HS	HS	
YPL129W	*TAF14*	General RNA polymerase II transcription factor	HS	HS		HS	HS			HS	
YDR080W	*VPS41*	Subunit of the homotypic vacuole fusion and vacuole protein sorting (HOPS) complex		HS	HS	HS				HS	HS
YOR106W	*VAM3*	Syntaxin-like vacuolar t-SNARE		HS	HS	HS				HS	HS
YGL212W	*VAM7*	Phosphatidylinositol-3-phosphate binding, SNAP receptor activity		HS	HS	HS				HS	HS
YGR104C	*SRB5*	Subunit of the RNA polymerase II mediator complex	R				HS		HS	HS	
YLR148W	*PEP3*	Component of vacuolar tethering complex that promotes vesicular docking/fusion	HS			HS		HS		HS	
YOR306C	*MCH5*	Riboflavin transporter		HS	HS					HS	
YJR118C	*ILM1*	ER and/or peroxisomal protein possibly involved in regulation fatty acid biosynthesis	HS	HS					HS	HS	
YJL175W	-	Dubious ORF	HS	HS			HS			HS	
YNL329C	*PEX6*	AAA-peroxin		R	R			HS [6]	HS		
YCR034W	*FEN1*	Fatty acid elongase	HS			HS		HS	HS		
YGL124C	*MON1*	Protein required for fusion of cvt-vesicles and autophagosomes with the vacuole		HS	HS	HS					HS
YDL077C	*VAM6*	Protein with a role in the tethering steps of vacuolar membrane fusion		HS		HS				HS	HS
YGR135W	*PRE9*	Subunit of the 20S proteasome	R				HS		HS		
YBL025W	*RRN10*	Subunit of upstream activation factor for RNA polymerase I		HS			HS		HS		
YJR104C	*SOD1*	Cytosolic superoxide dismutase		R			HS		HS		
YDR028C	*REG1*	Protein phosphatase type 1 regulator	R				HS			HS	
YMR202W	*ERG2*	C-8 sterol isomerase	R				HS			HS	
YLR056W	*ERG3*	C-5 sterol desaturase	HS	HS			HS			HS	
YML008C	*ERG6*	Delta(24)-sterol C-methyltransferase	R				HS			HS	
YER083C	*GET2*	Involved in insertion of proteins into the ER membrane	R				HS	HS			
YMR179W	*SPT21*	Protein with a role in transcriptional silencing	HS					HS	HS		
YHL033C	*RPL8A*	Protein L4 of the large (60S) ribosomal subunit	R					HS	HS		
YLR337C	*VRP1*	Actin-associated protein involved in cytoskeletal organization and cytokinesis	HS			HS		HS			
YPR153W	-	Unknown	R					HS		HS	
YDL142C	CRD1	Cardiolipin synthase	HS	HS				HS			
YMR064W	AEP1	Protein required for synthesis of the F1-F0 ATP synthase subunit Oli1p	HS	HS				HS			

YNL280C	*ERG24*	C-14 sterol reductase	R	R			HS				
YGL194C	*HOS2*	NAD-dependent histone deacetylase activity		R			HS				
YHL020C	*OPI1*	Transcriptional regulator of genes involed in lipid metabolism	R				HS				
YDR025W	*RPS11A*	Protein component of the small (40S) ribosomal subunit	R						HS		
YNL248C	*RPA49*	RNA polymerase I subunit A49	R						HS		
YMR124W	*EPO1*	Involved in septin-ER tethering	R	R	R						
YOR367W	*SCP1*	Binds and cross links actin filaments		R	R						
YLR372W	*SUR4*	Fatty acid elongase	HS			HS					
YBR255W	*MTC4*	Unknown, maintenance of telomere capping	R	R						HS	
YDR138W	*HPR1*	Subunit of THO/TREX complexes coupling transcription with mitotic recombination	R							HS	
YJL189W	*RPL39*	Protein component of the large (60S) ribosomal subunit	R							HS	
YPL193W	*RSA1*	Protein involved in the assembly of 60S ribosomal subunits	R	R [2]							
YKL009W	*MRT4*	Protein involved in mRNA turnover and ribosome assembly	R							HS	
YKR024C	*DBP7*	Putative ATP-dependent RNA helicase	R							HS	
YPR194C	*OPT2*	Pheromone-regulated protein proposed to be involved in mating	HS	HS						HS	
YPL260W	-	Putative substrate of cAMP-dependent protein kinase	R							HS	
YPL261C	-	Dubious ORF	R							HS	
YNL097C	*PHO23*	Regulation of expression		HS				HS			
YBL094C	-	Unknown		HS				HS			
YPL180W	*TCO89*	Subunit of TORC1	R					HS			
YHR191C	*CTF8*	Protein required for sister chromatid cohesion	R					HS			
YDR200C	*VPS64*	Protein required for cytoplasm to vacuole targeting of proteins	R					HS			
YMR143W	*RPS16A*	Protein component of the small (40S) ribosomal subunit	R					HS			
YCR081W	*SRB8*	Subunit of the RNA polymerase II mediator complex	R					HS			
YPL098C	*MGR2*	Subunit of the TIM23 translocase complex, required for growth of cells lacking the mitochondrial genome	HS					HS			
YHR155W	*YSP1/LAM1*	Putative sterol transfer protein	HS	HS							HS
YHR073W	*OSH3*	Member of an oxysterol-binding protein family	HS	HS							HS

[1] this study; [2] Kucejova et al. (2005), [3] Gustavsson et al. (2008); [4] Batova et al. (2010); [5] Ruggles et al. (2014); [6] Lockshon et al. (2007); [7] Barreto et al. (2011); [8] Hoepfner et al. (2014); (for full references see the main text); R, resistance; HS, hypersensitivity; Val, valinomycin; Nig, nigericin; Mon, monensin; CTBT, 7-chlorotetrazolo[5,1-c]benzo[1,2,4]triazine; PA, palmitoleic acid; OA, oleic acid; KD, cationic drugs (spermine, hygromycin B, and tetramethylammonium)
