# Supplementary material for: Identification of Yeast Mutants Exhibiting Altered Sensitivity to Valinomycin and Nigericin Demonstrate Pleiotropic Effects of Ionophores on Cellular Processes
Source: PLoS One. 2016 Oct 6;11(10):e0164175. doi: 10.1371/journal.pone.0164175 (PMC5053447; doi:10.1371/journal.pone.0164175)
Supplement: S6 Table — (PDF) [file pone.0164175.s009.pdf]

S6 Table. Staining of the control (wild-type) and ionophore-treated yeast cells using neutral red.

BY4741<br>[201 cells]	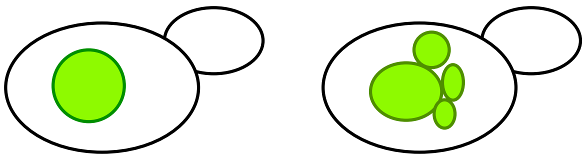<br>99.0%	
BY4741 + nigericin<br>[458 cells]		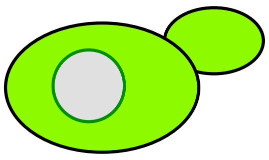<br>98.0%
BY4741 + valinomycin<br>[596 cells]	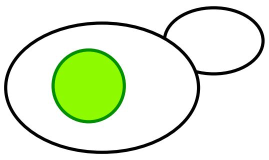<br>88.1%	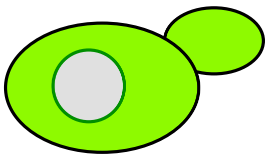<br>11.9%
